# Liver Pathology in Children with Diagnosed Inflammatory Bowel Disease—A Single Center Experience

**DOI:** 10.3390/jcm10225359

**Published:** 2021-11-17

**Authors:** Urszula Daniluk, Kamila Kwiatek-Sredzinska, Piotr Jakimiec, Jaroslaw Daniluk, Aleksandra Czajkowska, Dariusz Marek Lebensztejn

**Affiliations:** 1 Department of Pediatrics, Gastroenterology, Hepatology, Nutrition and Allergology, Medical University of Bialystok, 15-274 Bialystok, Poland; kamila.kwiatek90@gmail.com (K.K.-S.); piotrjakimiec89@gmail.com (P.J.); afilimoniuk@op.pl (A.C.); dariusz.lebensztejn@umb.edu.pl (D.M.L.); 2 Department of Gastroenterology and Internal Medicine, Medical University of Bialystok, 15-276 Bialystok, Poland; jaroslaw.daniluk@umb.edu.pl

**Keywords:** inflammatory bowel disease, Crohn’s disease, ulcerative colitis, hepatobiliary manifestations, children

## Abstract

Background: Inflammatory bowel disease (IBD) in children is frequently associated with liver pathology manifested as transient elevation of liver enzymes or specified liver diseases. The aim of the study was to evaluate the prevalence and the type of liver pathology in children with IBD within 2 years’ follow-up after the IBD diagnosis. Methods: We retrospectively reviewed records of children with IBD. Liver pathology was defined as elevated activity of liver enzymes (alanine transaminase (ALT) and/or gamma-glutamyl transpeptidase (GGT)) and bilirubin concentration in serum and/or as pathological changes of the organ on imaging tests (abdominal ultrasound and/or magnetic resonance cholangiopancreatography) or on liver histology performed when indicated. Results: Liver pathology was detected in 21 from 119 children (18%), including 7 (17%) with Crohn’s disease (CD) and 14 (18%) with ulcerative colitis (UC). Specified diagnosis for liver abnormality was found in 14 of 21 children (67%), including primary sclerosing cholangitis (PSC, 19%), non-alcoholic fatty liver disease (NAFLD, 19%), autoimmune sclerosing cholangitis (ASC, 5%), autoimmune hepatitis (AIH, 5%), cholelithiasis (5%), drug-induced liver disease (9%) and viral infection (herpes simplex virus, 5%). Most patients manifested mild IBD or were in clinical remission at the time of liver pathology diagnosis. 14% of patients with liver disease (including only cases with PSC) were diagnosed before IBD, 33% at the same time, and 52% in the later period. Patients with the specified diagnosis of liver pathology were younger, had higher ALT activity and more often demonstrated liver abnormalities on imaging tests. UC patients with idiopathic elevation of liver enzymes had higher pediatric ulcerative colitis activity index scores compared to children with specified liver disease. Conclusions: Liver pathology was observed in a significant percentage of children with IBD in our study. The majority of cases of hepatobiliary abnormalities were detected after diagnosis of IBD; therefore, children with IBD should undergo routine monitoring of liver enzymes.

## 1. Introduction

Inflammatory bowel diseases (IBD), such as Crohn’s disease (CD), ulcerative colitis (UC) and unclassified IBD are chronic diseases of unknown etiology, with periods of exacerbation and remission. Genetic and environmental factors, alterations of gut microbiota and metabolic pathways are involved in their pathogenesis [1,2,3,4,5]. Although IBD primarily affects the gastrointestinal tract, clinical manifestations also extend to other extra-intestinal systems and organs, such as the liver [6,7,8,9,10,11,12].

The hepatic dysfunction in IBD ranges from asymptomatic liver enzyme elevation to chronic liver disease [13]. Numerous studies regarding hepatobiliary manifestations in IBD in adults have been published [7,11,12,14,15,16,17]. However, data regarding the pediatric population are limited [6,8,9,18].

The primary aim of this study was to assess the incidence and type of liver pathology in children with IBD within two years of follow up diagnosis of IBD. The secondary aim was to compare some clinical and biochemical parameters, including markers of inflammation, and IBD activity scores between patients with and without liver involvement at the time of IBD diagnosis. We also evaluated whether there was a relationship between the type of liver pathology and IBD activity assessed at the onset of IBD diagnosis and at the time of the hepatobiliary tract disorder.

## 2. Materials and Methods

### 2.1. Patients

The retrospective study included children with newly diagnosed IBD from 2013–2018 in our department. Our department was the only center in the north-eastern part of Poland performing endoscopies in pediatric patients with suspected IBD in recent years. IBD was diagnosed on the basis of ESPGHAN guidelines [19]. Liver pathology was defined as elevated results of liver enzymes activity (alanine transaminase (ALT) and/or gamma-glutamyl transpeptidase (GGT)), and bilirubin concentration in serum according to the local laboratory reference ranges and/or as pathological changes of the organ on imaging tests, such as abdominal ultrasound (performed on all patients) and/or magnetic resonance cholangiopancreatography (MRCP), or on liver histology performed when indicated.

Demographic and clinical data were collected including IBD type and disease activity assessed using the pediatric Crohn’s disease activity index (PCDAI) and the Pediatric ulcerative colitis activity index (PUCAI), history of liver diseases, type of hepatobiliary manifestation and the relation of type of liver pathology to the IBD activity. Chronic liver diseases (primary sclerosing cholangitis (PSC), autoimmune sclerosing cholangitis (ASC), autoimmune hepatitis (AIH), non-alcoholic fatty liver disease (NAFLD)) were diagnosed in accordance with currently recommended criteria [20,21]. Patients with a suspicion of autoimmune liver disease underwent liver biopsy to confirm the diagnosis. The other appropriate tests were performed to exclude the viral infection, drug hepatotoxicity (diagnosed when the normalization of liver enzymes activity after drug discontinuation was achieved) or cholelithiasis.

### 2.2. Laboratory Tests Analysis

The biochemical analyses of the blood were performed in the local laboratory. ALT and GGT activities were determined by kinetic method (Cobas 6000-c501, Roche Diagnostics, Mannheim, Germany) and a serum ALT level >37 U/L and GGT values >40 IU/L were considered abnormal. Direct bilirubin was estimated using the Diazo Special method (Cobas 6000-c501, Roche Diagnostics, Mannheim, Germany) and its level >1 mg/dL was considered elevated. Selected markers of inflammation were determined in serum and stool based on the following methods. Faecal calprotectin (FC) concentration was determined by ELISA kit (IDK Calprotectin, Immundiagnostik, Bensheim, Germany). Erythrocyte sedimentation rate (ESR) was evaluated according to the Westergren method (Alifax Roler 20). Serum C-reactive protein (CRP) and albumin levels were determined by immunoturbodimetry (Cobas 6000-c501, Roche Diagnostics Mannheim, Germany). The complete blood count was measured using a Hematology Analyzer (Beckman Coulter). Ferritin was estimated by luminescent electrochemistry (Cobas e411, Roche Diagnostics Mannheim, Germany). Iron (Fe) was evaluated using colorimetric method (Cobas 6000-c501, Roche Diagnostics Mannheim, Germany). The cut-off values of the parameters were based on the local laboratory reference ranges.

### 2.3. Statistical Analysis

Categorical data were analyzed using the chi-square test and continuous variables were evaluated by the Student’s t test or the Mann–Whitney U test, depending on the normality of distribution (assessed by the Shapiro–Wilk test). A *p* value < 0.05 was considered statistically significant. Statistical analyses were performed using Statistica (Statsoft, Cracow, Poland).

## 3. Results

### 3.1. Comparison of Patients with and without Liver Involvement at the Time of IBD Diagnosis

Demographic and biochemical characteristics of the patients with and without liver pathology at the time of IBD diagnosis are listed in Table 1.

Among 119 patients (median age 14 years, 50 girls and 69 boys), enrolled to the study, 42 (35%) were diagnosed with CD and 77 (65%) with UC. Liver pathology was detected in 21 children (18%) with similar percentage in CD (17%) and UC (18%). We compared some clinical and biochemical parameters between patients with and without liver involvement at the time of IBD diagnosis (Table 1). Children with liver pathology had higher white blood cell (WBC) (*p* = 0.02) and platelet (PLT) (*p* = 0.04) counts compared to the rest of the patients. There were no statistically significant differences in age, sex, IBD type, IBD activity, levels of FC, ESR, CRP, haemoglobin (HGB), mean corpuscular volume (MCV), ferritin, Fe or albumin.

### 3.2. Characteristics of Patients with IBD Evaluated at the Time of Liver Disease Diagnosis

Clinical and biochemical features of 21 patients with IBD evaluated at the time of liver pathology diagnosis are presented in Table 2.

The etiology of liver pathology was confirmed in 14 patients (67%), mainly with UC (71%). All cases of PCS (*n* = 4), AIH (*n* = 1) and ASC (*n* = 1) were diagnosed only in children with UC. Moreover, NAFLD diagnosis was confirmed in patients mainly with UC (75%). Additionally, in IBD group with liver pathology, there was one case (5%) of cholelithiasis, two cases (9%) with thiopurine-induced hepatotoxicity and one (5%) with the herpes simplex virus (HSV) infection. Seventeen patients (81%) had abnormal ALT serum activity (median 76 U/L, range 44-805 U/L) with mild elevation (>1–2x the upper limit of normal (ULN)) in 47% of cases. The highest ALT values (>3x ULN) were found in children with known etiology of liver disease. Among them, AIH, ASC or PSC were diagnosed only in the UC group, but NAFLD or thiopurine-induced hepatotoxicity in CD cases. Eleven patients (52%) had elevated GGT serum activity (median 135 IU/L, range 49–418 IU/L) and the majority of them presented values greater than 3x ULN related to AIH, ASC and PSC diagnosis in only the UC group. The concomitant significant elevation of ALT and GGT was observed in four children with UC and AIH or ASC or PSC. Pathological changes on imaging tests such as liver enlargement or steatosis, or dilatation of common bile duct, or gallstones were found in twelve patients (57%), of whom one child had normal levels of liver enzymes. Hepatic abnormalities were diagnosed prior to IBD in 14% of cases (all with PSC), concomitant in 33% and thereafter in 52% of patients. The majority of children were in remission or demonstrated the mild form of IBD at the time of liver pathology diagnosis.

### 3.3. Comparison of Patients with Identified or Unidentified (Idiopathic) Liver Pathology

Having realized that the study group was small, we decided to investigate if there were differences between patients with diagnosed liver disease and unidentified liver disease. Clinical and biochemical features of patients with IBD and with coexisting identified or unidentified liver pathology are shown in Table 3.

Children with known liver disease were younger, had higher ALT activity and more often presented liver abnormalities on imaging tests compared to the patients with idiopathic liver disease. Patients with UC and idiopathic elevation of liver enzymes had higher PUCAI scores compared to children with specified liver disease. In children diagnosed with known liver disease, liver pathology was most often observed more than 6 months after IBD diagnosis, in contrast to children with idiopathic liver dysfunction, observed within or 1–3 months after IBD diagnosis. However, we realize the number of patients was small, thus the observed differences should be interpreted with caution.

## 4. Discussion

Liver pathology is common in patients with IBD and affects 11% to 40% of adults and 14% to 41.6% of children [6,7,8,9,11,12,14] depending on the study design. Our results showing 18% of children with detected liver abnormalities within two years of follow-up, are in line with published data. Hepatic disorders are reported in both UC and CD; however, usually they are more commonly related to patients with UC than with CD, as we also found in our study [9,11].

The association between IBD and elevated liver enzymes is not well established [7,11,13,15,17,22]. Abnormal liver function test results may be due to liver disease, a side effect of the therapy or an idiopathic origin [13]. Pusateri et al. showed that higher degree of ALT elevation (in particular >4× ULN) was significantly associated with a defined liver disease [6]. We also found the highest values of ALT (>3× ULN) in children diagnosed with known liver disease. Moreover, our study found that children with known liver disease were younger, had higher ALT activity and more often had liver abnormalities on imaging tests compared to the group with idiopathic liver pathology. In the study by Goyal et al. elevation of both ALT and GGT within 90 days after the diagnosis of IBD was associated with a markedly increased likelihood of IBD-associated liver disease [8]. In turn, Valentino et al. found that an elevated GGT (>252 U/L) suggested the presence of an underlying IBD-associated chronic liver disease [9]. Among our patients, the concomitant significant elevation of ALT and GGT was observed in children with UC and AIH or ASC or PSC.

Liver pathology may develop prior to, simultaneously or after IBD diagnosis. In the current study, hepatobiliary manifestations were detected before IBD in 14% of children (all with PSC), concomitant in 33% and thereafter in 52% of patients. Our results confirm the report of Silva et al., which showed 23.3% of cases diagnosed before IBD, 20% concurrently and 56.7% after diagnosis of IBD [12].

Markers preceding liver involvement in patients with IBD are unknown. The results of the present study revealed that pediatric patients with IBD and liver pathology had significantly higher WBC and PLT counts, both known but not specific markers of inflammation, than children without liver involvement [23]. Moreover, IBD activity scores did not vary between groups at the time of IBD diagnosis. On the other hand, when we evaluated all patients with IBD and concomitant liver pathology, 43% of cases were in remission of IBD and 24% had a mild form of the disease at the time of hepatobiliary manifestation, suggesting that IBD activity is not often related to liver disease.

Chronic liver diseases associated with IBD includes PSC, AIH, ASC, as well as drug-induced liver injury connected with medications used to treat IBD. PSC is a chronic inflammatory disorder that affects the intra- and extrahepatic biliary tree leading to bile duct and liver fibrosis. The diagnosis is based on typical bile duct lesions on cholangiography (20). PSC is the most common specific liver disease associated with IBD, especially with UC. Among pediatric patients with IBD, PSC is reported in 1.5% to 9.8% of cases [6,9,10,14,18]. The activity of PSC is independent of IBD activity [18]. PSC diagnosis may precede IBD, which results in earlier detection of IBD and, thus, lower disease activity [6,9,10,14,18]. In our study, PSC was found in four children (3%), all of whom with concomitant UC and three (75%) diagnosed with PSC before IBD.

AIH is a progressive inflammatory hepatopathy, the diagnosis is based on a combination of clinical, biochemical, immunological and histological features (20). The course of AIH associated with IBD is often complicated by periods of relapse and progression to cirrhosis [12]. Data show that the frequency of AIH in IBD ranges from 0.3% to 1.6%; a similar result (1%) was obtained in our study [13].

A clear distinction between AIH and PSC in the course of IBD may be difficult because of the number of overlapping features. ASC shares the diagnostic criteria for both PSC and AIH. ASC diagnosis is based on a clinical, biochemical, immunological and histological picture of AIH with typical cholangiography lesions of PSC (20). Valetino et al. found ASC in five of 300 children (1.7%) [9]. In our study, the ASC diagnosis was confirmed in only one patient with UC (1%).

Other common hepatobiliary diseases may coexist with IBD, comprising NAFLD, cholelithiasis and viral hepatitis. NAFLD, a common liver disease in patients with IBD in recent years, is not related to intestinal inflammation, but to overweight [10,12]. The widespread increase of obesity among children (also diagnosed with IBD) has made NAFLD a common pathology. According to the systemic review of Gizard et al., the prevalence of NAFLD in IBD patients ranges from 1.5% to 56% compared to 6.3% to 33% in the general population [10,13]. We found four children (3%) with NAFLD in our study group. The relationship between IBD and hepatic steatosis has been under observation [16,17,24]. Lipidomic studies have confirmed altered ceramide and sphingolipid content in children with IBD as well as with NAFLD, but interestingly, serum cholesterol and triglyceride levels in pediatric patients with IBD are comparable to the general population [3,4,25,26,27].

The association between cholelithiasis and IBD has been reported [13,16]. Suggested risk factors of gallstones in IBD are parenteral nutrition, changes in bile composition, ileal resection and impaired gallbladder emptying after surgery [28]. In adults, the prevalence rate of cholelithiasis was estimated from 11% to 34% of patients with CD. On the other hand, in patients with UC, it did not significantly differ from the general population [10]. In children with IBD, the presence of gallstones was described in 2.3% of cases [28]. Our study revealed a slightly lower frequency of cholelithiasis (1%).

Most drugs used in IBD are potentially hepatotoxic [12]. Pusateri et al. and Valentino et al. reported a significant association between the medications used for the treatment of IBD in children and elevated liver enzymes [6,9]. In the present study, two patients (2%) treated with 6-mercaptopurine developed thiopurine-induced hepatotoxicity. Immunosuppressive therapies used in IBD predispose to viral infections, which may affect the liver. In our study, one child (1%) suffered from viral infection caused by the herpes simplex virus.

The lack of a specific cause for the elevated liver enzymes in patients with IBD is frequently observed [6,9,10]. This problem occurred in 87% of children with IBD in a retrospective study by Pusateri et al. [6]. Our analysis showed lower frequency of idiopathic liver pathology (33%). The pathogenesis of this phenomenon is unknown. Changes in microbiome connected with IBD can affect liver function through products of bacterial metabolism, such as ethanol or activation of immune cells [29,30]. It has been suggested that active IBD may also be an etiology for elevated liver enzymes; however, the available data are inconsistent. Some studies have reported a correlation between elevated liver enzymes and disease activity, but others have not shown any association [7,11,15]. In our study, children with UC and idiopathic elevation of liver enzymes had significantly higher diseased activity scores assessed by PUCAI than patients with known etiology of liver disease. It is possible, that in cases of idiopathic increase in liver enzymes coexisting with moderate/severe IBD, the elevated liver enzymes could be related to IBD activity, as an extraintestinal manifestation.

Limitations of our study are mainly related to the retrospective character of the design, which predispose to loss of some data. In addition, our study group was relatively small, especially for those with liver pathology; therefore, our results should be interpreted with caution and not extrapolated to all pediatric patients with IBD. More long-term and prospective studies with a larger group of patients are needed to confirm our results. The strength of the present study is that our department is the reference pediatric endoscopic center offering histopathological examinations in the north-eastern part of Poland. We may assume that all children diagnosed with IBD represent the entire pediatric population with IBD from this region.

## 5. Conclusions

Liver pathology was found in a significant percentage of children with IBD in our study. Higher ALT and GGT activities were significantly associated with defined liver pathology; however, in many cases, idiopathic increases in liver enzymes have been observed. Diagnosis of PSC, AIH, ASC was more common in UC patients, but other liver pathologies such as NAFLD, cholelithiasis, drug hepatotoxicity or viral infections were found in both IBD groups. Most patients manifested a mild form of IBD or were in remission at the time of hepatobiliary manifestations. The majority of cases of liver pathology were detected after diagnosis of IBD, therefore children with IBD should undergo routine monitoring of liver enzymes.

## Figures and Tables

**Table 1:** Demographic and biochemical parameters of patients with and without liver pathology, collected at the time of the IBD diagnosis.

Parameter	Patients with Liver Pathology (*n* = 21)	Patients without Liver Pathology (*n* = 98)	*p*
Age, median (range), years	14 (3–17)	14 (1–17)	NS
Sex, no. of female (%)	9 (43%)	41 (42%)	NS
IBD type, *n* (%)			
CD	7/42 (17%)	35/42 (83%)	NS
UC	14/77 (18%)	63/77 (82%)	NS
IBD activity, median (range), score			
PCDAI	35 (5–42.5)	25 (5–62.5)	NS
PUCAI	45 (10–75)	35 (5–80)	NS
IBD activity, *n* (%)			
Remission	1 (5%)	2 (2%)	NS
Mild	7 (33%)	45 (46%)	NS
Moderate	11 (52%)	41 (42%)	NS
Severe	2 (10%)	10 (10%)	NS
FC (ug/g), median (range)	2127.1 (88–2874)	1689.2 (18–3479)	NS
ESR (mm/h), median (range)	34 (3–120)	19 (2–130)	NS
CRP (mg/L), median (range)	13 (0.1–141.2)	5.5 (0.01–299)	NS
WBC (10^3^/µL), median (range)	10.31 (4.87–28.7)	8.06 (3.42–20)	0.02
PLT (10^3^/µL), median (range)	409 (210–676)	329 (75–807)	0.04
HGB (g/dL), median (range)	11.8 (8.7–14.9)	12.2 (8.5–15.8)	NS
MCV (fl), median (range)	77.2 (68.7–89)	78.9 (62.7–89.6)	NS
Ferritin (ng/mL), median (range)	21 (4.83–127.2)	32.75 (2.12–462.7)	NS
Fe (ug/dL), median (range)	36 (11–91)	36 (7–155)	NS
Albumin (g/dL), median (range)	4.16 (2.17–5.01)	4.36 (2.78–5.36)	NS

NS—nonsignificant, IBD—inflammatory bowel disease, CD—Crohn’s disease, UC—ulcerative colitis, PCDAI—pediatric Crohn’s disease activity index, PUCAI—pediatric ulcerative colitis activity index, FC—faecal calprotectin, ESR—erythrocyte sedimentation rate, CRP—C-reactive protein, WBC—white blood cells, PLT—platelets, HGB—haemoglobin, MCV—mean corpuscular volume, Fe—iron.

**Table 2:** Clinical and biochemical parameters of patients with IBD evaluated at the time of the liver pathology diagnosis.

Parameter	Patients with Liver Pathology (*n* = 21)		
Type of hepatobiliary manifestation, *n* (%):	IBD (*n* = 21)	CD (*n* = 7)	UC (*n* = 14)
No defined etiology (idiopathic)	7 (33%)	3 (43%)	4 (29%)
PSC	4 (19%)	0	4 (29%)
ASC	1 (5%)	0	1 (7%)
AIH	1 (5%)	0	1 (7%)
NAFLD	4 (19%)	1 (14%)	3 (21%)
Cholelithiasis	1 (5%)	1 (14%)	0
Drug hepatotoxicity	2 (9%)	1 (14%)	1 (7%)
Viral infection	1 (5%)	1 (14%)	0
IBD activity, median (range), score			
PCDAI	NA	5 (2.5–15)	NA
PUCAI	NA	NA	37.5 (0–70)
IBD activity, *n* (%)			
Remission	9 (43%)	5 (24%)	4 (19%)
Mild	5 (24%)	2 (9%)	3 (14%)
Moderate	6 (28%)	0	6 (28%)
Severe	1 (5%)	0	1 (5%)
Time of liver pathology diagnosis in relation to IBD diagnosis, *n* (%)			
Preceding	3 (14%)	0	3 (14%)
Concomitant	7 (33%)	0	7 (33%)
After	11 (52%)	7 (33%)	4 (19%)
○ 1–3 months	3 (27%)	3 (43%)	0
○ 4–6 months	0	0	0
○ more than 6 months	8 (73%)	4 (57%)	4 (100%)
Increase of ALT, *n* (%); median U/L (range)	17 (81%); 76 (44–805)	5 (24%); 76 (67–135)	12 (57%); 72 (44–805)
>1–2 × ULN	8 (47%); 58 (44–70)	2 (12%); 68.5 (67–70)	6 (35%); 52 (44–68)
>2–3 × ULN	3 (18%); 76 (76–86)	1 (6%); 76	2 (12%); 81 (76–86)
>3 × ULN	6 (35%); 137 (112–805)	2 (12%); 130.5 (126–135)	4 (24%); 262 (112–805)
Increase of GGT, *n* (%); median IU/L (range)	11 (52%); 135 (49–418)	3 (14%); 60 (56–255)	8 (38%); 201.5 (49–418)
>1–2 × ULN	5 (45%); 56 (49–61)	2 (67%); 58 (56–60)	3 (38%); 51 (49–61)
>2–3 × ULN	0	0	0
>3 × ULN	6 (55%); 274 (135–418)	1 (33%); 255	5 (62%); 280 (135–418)
Increase of ALT and GGT > 3 × ULN	4 (19%)	0	4 (19%)
Elevated direct bilirubin, *n* (%)	3 (14%)	2 (9%)	1 (5%)
Liver abnormalities in imaging studies, *n* (%)	12 (57%)	2 (9%)	10 (48%)

NA—nonapplicable, IBD—inflammatory bowel disease, CD—Crohn’s disease, UC—ulcerative colitis, PSC—primary sclerosing cholangitis, ASC—autoimmune sclerosing cholangitis, AIH—autoimmune hepatitis, NAFLD—non-alcoholic fatty liver disease, PCDAI—pediatric Crohn’s disease activity index, PUCAI—pediatric ulcerative colitis activity index, ALT—alanine transaminase, GGT—gamma-glutamyl transpeptidase, ULN—upper limit of normal.

**Table 3:** Comparison of IBD patients’ parameters with coexisting identified liver pathology or idiopathic liver pathology.

Parameter	Patients with Identified Liver Pathology (*n* = 14)	Patients with Idiopathic Liver Pathology (*n* = 7)
Age, median (range), years	11.5 (3–16)	16 (10–17)
Sex, no. of female (%)	6 (43%)	3 (43%)
IBD type, *n* (%)		
CD	4 (29%)	3 (43%)
UC	10 (71%)	4 (57%)
IBD activity at the time of liver pathology diagnosis, median (range), score		
PCDAI	5 (2.5–10)	5 (5–15)
PUCAI	25 (0–60)	57.5 (55–70)
IBD activity at the time of liver pathology diagnosis, *n* (%)		
Remission	7 (50%)	2 (29%)
Mild	4 (29%)	1 (14%)
Moderate	3 (21%)	3 (43%)
Severe	0	1 (14%)
Time of liver pathology diagnosis in relation to IBD diagnosis, *n* (%)		
Preceding	3 (21%)	0
Concomitant	3 (21%)	4 (57%)
After	8 (57%)	3 (43%)
○ 1–3 months	0	3
○ 4–6 months	0	0
○ more than 6 months	8	0
ALT (U/L), median (range)	73 (32–805)	44 (20–86)
GGT (IU/L), median (range)	44 (14–418)	49 (13–135)
WBC (10^3^/µL), median (range)	10.66 (6.11–28.7)	9.75 (4.87–20)
PLT (10^3^/µL), median (range)	422.5 (308–547)	312 (210–676)
Liver abnormalities in imaging studies, *n* (%)	11 (79%)	1 (14%)

NS—nonsignificant, IBD—inflammatory bowel disease, CD—Crohn’s disease, UC—ulcerative colitis, PCDAI—pediatric Crohn’s disease activity index, PUCAI—pediatric ulcerative colitis activity index, ALT—alanine transaminase, GGT—gamma-glutamyl transpeptidase, WBC—white blood cells, PLT—platelets.

## Data Availability

Data is contained within the article.
